# Lipid-Regulating Effect of Traditional Chinese Medicine: Mechanisms of Actions

**DOI:** 10.1155/2012/970635

**Published:** 2012-05-07

**Authors:** Wei-Jian Bei, Jiao Guo, Hai-Yun Wu, Yang Cao

**Affiliations:** ^1^Key Unit of Modulating Liver to Treat Hyperlipidemia of SATCM, State Administration of Traditional Chinese Medicine (SATCM) Level 3 Lab of Lipid Metabolism, Guangdong TCM Key Laboratory for Metabolic Diseases, Institute of Chinese Medicinal Sciences, Guangdong Pharmaceutical University, Guangzhou University of Chinese Medicine, Guangzhou Higher Education Mega Centre, Guangzhou 510006, China; ^2^Institute of Geriatric Cardiology, Chinese PLA General Hospital, Beijing 100853, China

## Abstract

Traditional Chinese medicine (TCM) has been increasingly used for the treatment of dyslipidemia and cardiovascular disease. Recently, much progress has been made in studies on the mechanisms of action of the lipid-regulating effect of TCM in animal experiments. Current researches showed that the lipid-regulating effect of TCM may be related to the following actions: (1) inhibiting intestinal absorption of lipids; (2) reducing the biosynthesis of endogenous lipids; (3) increasing the catabolism of lipid, sterol substances in live system; (4) increasing the secretion of sterol substances in live system; (5) regulating transcription factors related to lipid metabolism. This paper provides an overview of the recent advances and discusses their implications in future development of lipid-lowering drugs from TCM.

## 1. Introduction

Dyslipidemia refers to a disruption of lipid metabolism with exceeding serum levels of cholesterol (TC), triglyceride (TG), low-density lipoprotein-cholesterol (LDL-C), and/or lower level of high-density lipoprotein-cholesterol (HDL-C). Serum levels of lipids and lipoprotein lipids are among the most potent and best substantiated risk factors for atherosclerotic diseases, particularly coronary heart disease (CHD) [1].

In human, lipids homeostasis is regulated by well-balanced mechanisms of intestinal uptake, endogenous synthesis and metabolism, transport in lipoprotein particles, and biliary excretion. The metabolism of cholesterol and fatty acids and their associated lipid transport particles (the lipoproteins) occurs in the gut, liver, and the peripheral tissues. Drugs that alter lipids concentration act mainly by altering the kinetics of one or more parts of the metabolic cycle.

Traditional Chinese medicine (TCM) has been increasingly used for the treatment of dyslipidemia and cardiovascular disease. In a previous paper, we have reviewed the efficacy of TCM for the treatment of dislipidemia, which has been confirmed by numerous clinical studies as well as laboratory researches [2].

In this paper, we will focus on the underlying mechanisms of actions of the lipid-regulation effect of TCM.

## 2. Inhibiting the Intestinal Absorption of Cholesterol

The absorption of dietary lipids (cholesterol, fatty acids, phospholipids, etc.) in the intestine is an important source of serum lipids. Cholesterol absorption is a key regulatory target in human lipid metabolism because it determines the amount of endogenous biliary as well as dietary cholesterol that is retained, thereby influencing cholesterol balance. Cholesterol in the intestinal tract is derived from the diet and bile. Whereas dietary intake ranges from <50 mg/day (pure vegetarians) to 750 mg/day, biliary cholesterol input is 3 to 10 times higher and ranges from 500 to 2,400 mg/day.

Plant sterols (e.g., stigmasterol and *β*-sitosterol) reduce serum LDL cholesterol level by competitively inhibiting intestinal cholesterol absorption. Recent findings also suggest that plant stanols/sterols actively influence cellular cholesterol metabolism within intestinal enterocytes, and, in response to the reduced supply of exogenous cholesterol, receptor-mediated lipoprotein cholesterol uptake is probably enhanced, as shown by increased LDL receptor expression [3].

Plant sterols are rich in a variety of traditional Chinese herbal medicine, such as fleece-flower root, cassia seed, eucommia, rhubarb, polygonum, and turmeric. In addition, cellulose, pectin, and agar, which are also rich in many Chinese herbal medicines, can reduce the absorption of cholesterol by forming a complex with cholate to impede the formation of cholesterol microparticles in the intestine [3, 4].

An *in vivo* experiment showed that the addition of 0.5% Fr2-3 (a tea leaf fraction containing 72% saponins with high *in vitro* antihypercholesterolemic activity) to a high-cholesterol diet suppressed the increase in serum cholesterol levels in rats. Fr2-3 induced a decrease in the liver cholesterol and triglyceride levels and an increase in the fecal excretion of cholesterol [4].

Anthraquinones in fleece-flower root inhibit absorption of cholesterol by increasing peristalsis in the intestine, enhance the converting of cholesterol into the bile acid, and therefore increase the cholesterol excretion via the bile [5]. Lecithin in fleece-flower root can also prevent the cholesterol deposition in the liver, increase the transportation and converting of cholesterol, thus reduce the concentration of cholesterol in serum and liver, and prevent its infiltrating into artery intima [5]. Furthermore, lecithin increases the activity of the cholesteryl esterase in vessel wall and inhibits the activity of Acyl coenzyme A-cholesterol acyltransferase (ACAT) [5]. Components in cassia proteins were reported to combine with bile acid and inhibit the absorption and aggradations of cholesterol in the body, leading to the decrease of serum level of TC, TG, and LDL in the hyperlipidemic rats [6].

Recent insights in the role of ATP-binding cassette (ABC) transporters ABCG5 and ABCG8, as well as the identification of Niemann-Pick C1 Like 1 (NPC1L1) protein as sterol transporter in the gut, focused attention on sterol transport processes in the small intestine and the liver [7–9]. NPC1L1 is the target of cholesterol absorption inhibitor Ezetimibe, which prevents NPC1L1 entering cells by avoiding NPC1L1 incorporated into vesicles, reducing the uptake of cholesterol by cells, impending the cholesterol absorption in intestine, and thus reducing the serum cholesterol concentrations significantly [10]. To date, no research has been reported on the effects of TCM on NPC1L1. We believe that screening TCM with suppressing activities on NPC1L1 will lead to the discovery of new components and new drugs for regulating cholesterol absorption in intestine and the new drugs of lipid lowering.

ACAT plays an important role in the absorption, transport, and storage of cholesterol by catalyzing the cholesterol and long-chain fatty acid to form cholesterol ester *in vivo*. Inhibition of ACAT may reduce the levels of plasma TC and LDL cholesterol, reduce the accumulation of cholesterol ester on the arterial wall, and prevent the formation of atherosclerosis. Hawthorn triterpene acids were reported to reduce blood TC by inhibiting the activation of ACAT in the hamster intestinal. Oleanolic acid (OA) and ursolic acid (UA) are responsible for the cholesterol-lowering effect of hawthorn by inhibiting intestinal ACAT activity. In addition, hawthorn and particularly its bioactive compounds (OA and UA) enhanced the cholesterol-lowering effect of plant sterols [11].

The microsomal triglyceride transfer protein (MTP) plays a pivotal role in the assembly and secretion of apolipoprotein B. Fresh garlic extract (FGE) at 3~6 g/L reduced MTP mRNA levels in both the human hepatoma HepG2 and intestinal carcinoma Caco-2 cells in dose-dependent fashion; maximal reductions reached to 72% and 59%, respectively. Rats fed FGE had significantly (46% of the control) lower intestinal MTP mRNA levels compared with the control rats. Long-term dietary supplementation of fresh garlic may exert a lipid-lowering effect partly through reducing intestinal MTP gene expression, thus suppressing the assembly and secretion of chylomicrons from intestine to the blood circulation [12].

## 3. Inhibiting the Endogenous Lipid Biosynthesis

Cells *in vivo* synthesize cholesterol via the mevalonate or HMG-CoA reductase (HMGCR) pathway. HMGCR and 7-dehydrocholesterol reductase (DHCR7) are the key rate-limiting enzymes of cholesterol synthesis and play vital roles in maintaining the cholesterol homeostasis. High-energy, high-fat, and high-saturated fatty acid diet, which can promote the synthesis of cholesterol, is the most common risk factor of hyperlipidemia, especially hypercholesterolemia.

Dozens of TCM herbs, including coptis (berberine), salvia, hawthorn, hawthorn-flavone, green tea and its active ingredient-catechol, triterpenoid in alisma, and gypenosides, have been reported to inhibit the endogenous synthesis of cholesterol [5].

Chang reported that berberine might inhibit the synthesis of cholesterol in the liver by upregulating the expression of hepatic Insig-2 gene at low dose, while at high dose, berberine reduces the expression of Insig-2 mRNA and protein [13].

*Astragalus* and *Angelica* were also reported to reduce the serum level of TC and LDL-C via inhibiting HMGCR activity in hyperlipidemic rat. Angelica and ferulic acid competed with 5-methyl acid pyrophosphate and inhibit the activity of methyl valerate-5-pyrophosphate decarboxylase of the rat liver cell and thereby inhibit the cholesterol biosynthesis [14].

High-dose aqueous extracts from artichoke leaves were found to inhibit cholesterol biosynthesis from 14C acetate in primary cultured rat hepatocytes in a concentration-dependent biphasic manner with moderate inhibition (approximately 20%). Cynaroside and particularly its aglycone luteolin in the artichoke extract were mainly responsible for inhibition of hepatic cholesterol biosynthesis in an indirect downmodulation of HMGCR, which may contribute to the recently confirmed hypolipidemic effect in human [15].

Fatty acid synthase (FAS) catalyzes the last step of fatty acid biosynthesis. Radix Notoginseng can inhibit FAS and thus lower serum TG level. Zhang reported that *Panax notoginseng* saponins [16] and green tea polyphenols [17, 18] have been reported to inhibit the mRNA and protein expression of I*κ*-B*α* and FAS in abdominal aortic tissue or in adipocyte, so as to lower lipid in high-fat hyperlipidemic rat.

## 4. Regulating Lipoprotein Lipase Activity

Lipoprotein lipase (LPL) and hepatic lipase (HL) play vital roles in the metabolism of chylomicrons and very low-density lipoprotein. Lacking of these two enzymes or their dynamic abnormalities might lead to dyslipidemia, metabolic syndrome, atherosclerosis, diabetes, preeclampsia, and other diseases [19, 20].

Increase in mRNA and protein levels of LPL might increase the activity of LPL in the adipose tissue and plasma, promote the clearance of VLDL and postprandial plasma lipid, reduce plasma triglycerides, increase HDL-C levels, and therefore prevent hypercholesterolemia induced by high fat diet and development of atherosclerosis [21, 22].

Berberine, hawthorn, turmeric, red yeast, rhubarb, and purslane might increase the expression and activity of the LPL [5, 23–26]. Upregulating the transcription of LPL mRNA and LDL-R-mRNA in liver may also be one of the molecular mechanisms of blood lipids regulation of Xuezhikang, the red yeast extract [27]. Red ginseng acidic polysaccharide (RGAP), isolated from Korean red ginseng, was also reported to significantly enhance the serum activity of LPL [28].

Hepatic lipase (HL) is another important glycoprotein that catalyzes the hydrolysis of lipoprotein triacylglycerols and phospholipids. The majority of HL is synthesized and secreted by the liver and bound to heparin sulfate proteoglycans on the surface of sinusoidal endothelial cells and external surfaces of microvilli of parenchymal cells in the space of Disse, promoting the uptake of HDL and apolipoprotein-B-containing remnant particles [21, 29, 30]. Its catalytic activity contributes to the remodeling of LDL and high-density HDL to smaller, denser particles. HL also participates, with surface proteoglycans, the scavenger receptor B1 (SR-B1) and the LDL receptor-like protein, as a ligand in promoting hepatic uptake of lipoproteins. Recent *in vivo* and *in vitro* studies suggest alternative pathways, both through its catalytic activity and, independently, by which HL may modulate the development of cardiovascular and cerebrovascular disease [29, 30].

Saponins from *Tribulus terrestris* increase the activity and expression of HL in liver and the activity of LPL in skeletal muscle. These might be, at least in part, the underlying mechanisms for its effect in reducing serum levels of TG and TC [31].

Studies in our laboratory showed that Fufang Zhenzhu Tiaozhi Fang (FTZ) could improve serum lipid profile (TC, TG, apoB, LDL-C and HDL-C, apoA). Experimental studies in rat demonstrated that FTZ upregulates LPL and HL expression and increases the activity of both LPL and HL [32].

*Pleurotus eryngii *water extract (PEE) showed significant inhibitory activity against pancreatic lipase by preventing interactions between lipid emulsions and pancreatic lipase *in vitro*. The hypolipidemic effect of PEE in fat-loaded mice may be due to low absorption of fat caused by the inhibition of pancreatic lipase [33]. The ethyl acetate fraction of the rhizome of *Alpinia officinarum* (AO) exhibited potent inhibition of pancreatic lipase. 3-Methylethergalangin was isolated from the fraction as an inhibitor of pancreatic lipase with an IC50 value of 1.3 mg/mL (triolein as a substrate). AO and its ethyl acetate fraction significantly lowered the serum TG level in corn oil feeding-induced triglyceridemic mice, and serum TG and cholesterol in Triton WR-1339-induced hyperlipidemic mice. The hypolipidemic activity of AO and 3-methylethergalangin was due to the inhibition of pancreatic lipase [34].

The ethanolic extract of *Ananas comosus* L. leaves (AC) (0.40 g/kg) significantly reduced the increased serum triglycerides by 40% in fructose-fed mice. AC also significantly inhibited serum TC in Triton WR-1339 and alloxan plus high-fat diets-induced hyperlipidemic mice. AC (0.01–100 *μ*g/mL) selectively activated LPL activity by 200%–400% and significantly inhibited HMGCR activity by 20%–49% *in vitro*. Furthermore, AC (0.40 g/kg) did no increase mice liver weights as fenofibrate (0.20 g/kg) administration. Xie et al. recognized that AC will be a new potential natural product for the treatment of hyperlipidemia through the mechanisms of inhibiting HMGCR and activating LPL activities, which was different from those with fibrates but may be partly similar to those with statins. It is hopeful that AC may serve as the adjuvant for fibrates [35]. 

## 5. Regulation of Cholesterol Transport

Inhibiting plasma cholesterol ester transfer protein (CETP) might increase the content of HDL-C and ApoA1, which is helpful to reduce TC, TG, and LDL-C. Lin et al. used a software to select CEPT inhibitor fictitiously. Dihydrotanshinone I, chosen as the target molecule with the CEPT-inhibiting active ingredient from salvia, was found to increase HDL and decrease LDL levels and could reduce TC and TG levels in serum and liver in the experimental hypercholesterolemic rat [39].

Berberine, an alkaloid isolated from the Chinese herb *Coptis chinensis*, has been recently identified as a new cholesterol-lowering drug and reduced serum TC and LDL-C levels in hyperlipidemia rats in a dose-dependent manner. The LDL cholesterol-lowering effect of berberine was attributed to its activity on hepatic LDLR expression via a new mechanism distinct from that of statins [36–38]. In a human hepatoma cell line (HepG2) as well as in hyperlipidemic hamsters, Kong et al. showed that berberine upregulated the expression of LDLR through stabilization of its mRNA involving an extracellular-regulated-kinase- (ERK-)dependent mechanism [36].

Vijayakumar reported that hypolipidemic effect of a novel thermostable extract of Fenugreek seeds (*Trigonella foenum-graecum,* TEFS) was due to inhibition of fat accumulation and upregulation of LDLR. Under sterol-enriched condition, TEFS upregulated low-density lipoprotein receptor (LDLR) expression resulting in enhanced LDL uptake. TEFS administration for 15 days decreased the elevated serum TG, LDL-cholesterol, and body weight in a dose- and time-dependent manner in fat-supplement-fed C57BL6/J mice [42].

## 6. Promotion of Cholesterol Converting into Bile Acid and Excreting

Cholesterol is transformed into bile acids and excreted from the digestive tract under the catalysis of liver cholesterol 7*α*-hydroxylase (CYP7A1). About 2/5 synthesized cholesterol is converted into bile acids, under the catalysis of CYP7A1. Promoting CYP7A1 activity might enhance cholesterol to convert into bile acid, thereby to remove the cholesterol from the body, which is the primary mechanism for maintaining in cholesterol homeostasis [53, 54].

Xue et al. reported that radix *Salviae miltiorrhizea* extract containing danshensu is absorbed into blood after oral administration. Sodium danshensu (25~200 *μ*g/mL) increased the percentage of cell viability in experiment of amphotericin B cell model and upregulated the expression of CYP7A mRNA in BRL cells, indicating that Danshensu can inhibit the synthesis of endogenous cholesterol and increase the expression of CYP7A1 mRNA to promote cholesterol transformation into bile acid in hamster liver cells [40, 41].

Zhang et al. reported that hawthorn fruit aqueous ethanolic extract decreased serum TC and TG by 10% and 13%, respectively, in hamsters which were fed with semisynthetic diet containing 0.1% cholesterol. Compared with the control, hawthorn fruit led to greater excretion of both neutral and acidic sterols. Further enzymatic assays showed that hawthorn fruit might promote the excretion of bile acids by upregulation of hepatic CYP7A1 activity and inhibition of cholesterol absorption mediated by downregulation of intestinal acyl CoA cholesterol acyltransferase (ACAT) activity [24].

Garcia-Diez reported that the addition of pectin to the diet resulted in lower serum and liver cholesterol concentrations (−27% and −17%, resp.) in male Wistar rats fed a fiber-free or a pectin-supplemented (7 g/100 g) diet for 4 wk. Fecal bile acid excretion (+168%) and the hepatic activity of CYP7A1 (+70%) were significantly higher in pectin-fed animals. HMGCR activity was also significantly greater (+11%) in the presence of dietary pectin. Pectin may increase hepatic synthesis of bile acids and liver depletion of cholesterol in rats, resulting in a higher rate of cholesterol synthesis and reduced serum cholesterol concentrations [46].

Vergara-Jimenez reported that plasma LDL cholesterol, TG, apolipoprotein B, and hepatic cholesteryl ester were lower in guinea pigs fed pectin (PE) and psyllium (PSY) compared to the control group. In addition, a 45% higher number of hepatic apoB/E receptors were observed by PE and PSY intake. Hepatic ACAT, HMGCR, and CYP7A1 activities were higher in the high-fat (HF) compared to the low-fat (LF) groups. PSY intake with HF resulted in upregulation of CYP7A1 and HMGCR activities. ApoB secretion was reduced by pectin and psyllium intake, while LDL fractional catabolic rates were 100% faster in guinea pigs fed PE or PSY in the HF groups [47].

Psyllium, the husks from *Plantago ovata* (PO), is recognized as a potent agent in lowering plasma cholesterol. Plasma triglycerides and LDL cholesterol were 34% and 23%, respectively, lower in the PO groups compared with the control male Hartley guinea pigs. Lecithin cholesterol acyltransferase (LCAT) and cholesterol ester transfer protein (CETP) activities were significantly affected by the PO diets. The control group had 100% and 36% higher LCAT and CETP activities, respectively, compared with the PO groups. Hepatic cholesteryl ester concentrations were 50% lower in the PO groups compared with the control. The activity of HMGCR was upregulated in the PO groups by 37%. Similarly, the activity of CYP7A1 was 33% higher in the PO groups. Fecal bile acids were 3 times higher in the PO groups than in the control group. PO exerts its hypolipidemic effect by affecting bile acid absorption and altering hepatic cholesterol metabolism [48].

FTZ significantly decreased the levels of serum TC, TG, and LDL-C whilst elevated the serum HDL-C and decreased serum atherogenic index (A.I.) values in high-lipid-diet-induced hyperlipidemic rats. Furthermore, FTZ showed significant antihyperlipidemic effect by at least three pathways in the high-lipid diet-induced-hyperlipidemic rats: (1) upregulating the gene expression and activity of CYP7A1 which promotes the conversion of cholesterol into bile acid; (2) downregulating the gene expression and activity of HMGCR to reduce *de novo* synthesis of cholesterol; (3) increasing the cholesterol excretion from feces. In these three pathways, HMGCR and CYP7A1 are two pivotal enzymes in lipid cholesterol metabolism and are expressed mainly in hepatic cells, which support the new TCM treatment strategy: modulating liver to treat hyperlipemia [49].

## 7. Effects of TCM on Regulation of Transcription Factors Related to Lipid Metabolism

Metabolic enzymes and receptors involved in the lipid metabolism are subject to positive and negative regulation of dozens of transcription factors. At present, some of intensively investigated genes involved in the lipid metabolism are peroxisome proliferator-activated receptor (PPARs) [55, 56], sterol regulatory element binding protein (SREBPs) [57, 58, 60, 61], and liver receptor (the liver X receptors, LXR) [59–61] gene. In recent years, there have been increasing studies to explore the regulating effects of TCM on transcription factors related to lipid metabolism.

### 7.1. PPARs Regulation

Studies have showed that several active ingredients from TCM herbs are PPARs agonists, and their active ingredient can activate PPARs.

Coptis and its active ingredient berberine could activate the PPARs, including PPAR*α* [50] and PPAR-*γ* [23]. Hawthorn flavonoids might activate PPAR-*α* and increase the LPL activity of the blood and muscle tissue of hyperlipidemic rats or mice [24]. Curcumin could activate PPAR-*γ* and thus increase LPL activity in tissues and blood [25, 26].

*Gynostemma pentaphyllum* has showed antihyperlipidemic and hypoglycemic effects in the obese Zucker fatty diabetic rat model. Its active ingredient gypenoside XLIX was proved as a naturally occurring PPAR*α* activator [43].

Gao et al. found that oleanolic acid extracted from *Ligustrum lucidum* could lower TC, TG, and LDL-C by activating the gene expression of cell PPAR-*α*, *γ* and protein kinase B [51]. Ginseng can also lower serum lipid by activating PPAR-*α* in cells [45].

### 7.2. SREBP Regulation

Lipid homeostasis is subject to the regulation of SREBPs, a class of membrane-bound transcription factor [57, 58, 60, 61].

Berberine promotes the translation of LDL-R gene by directly stabilizing the structure of SRE1 of LDL-R gene 5′ flanking region [36].

TEFS inhibited accumulation of fat in differentiating and differentiated 3T3-L1 cells via decreased expression of adipogenic factors such as PPAR-*γ*, SREBP-1, and CAAT element-binding proteins-alpha (c/EBP-alpha). TEFS treatment also significantly decreased cellular TG and cholesterol concentrations in HepG2 cells via reduced expression of SREBP-1, at mRNA as well as protein level [42].

Morroniside significantly decreased the elevated serum TG and alanine aminotransferase levels as well as hepatic glucose and lipids contents in a dose-dependent manner in type 2 diabetes model mice (C57BLKS/J db/db mice). The administration of morroniside also increased the antioxidative effects in the liver of db/db mice with hyperglycemia and dyslipidemia. The elevated expressions of nuclear factor-kappa Bp65, cyclooxygenase-2, inducible nitric oxide synthase, SREBP-1, and SREBP-2 were downregulated in the liver of db/db mice, but significantly increased PPARa expression by morroniside [52].

### 7.3. LXRs Regulation by TCM

LXRs are the receptors of nuclear-oxidized steroid. [59–61]. LXRs were activated by inducing SREBP-1c and finally upregulated the genes involved in fatty acid and triglyceride synthetic genes and fatty acid synthase, such as FAS, SCD1. At the same time, LXRs were also involved in regulating insulin-induced SREBP-1c gene expression [62, 63].

The plant sterol guggulsterone [4, 17 (20)-pregnadiene-3,16-dione] (GS) is the active substance in guggulipid, the resin of the guggul tree (*Commiphora mukul*) used to treat a variety of disorders in humans, including dyslipidemia, obesity, and inflammation. GS is a highly efficacious antagonist of the farnesoid X receptor (FXR), a nuclear hormone receptor that is activated by bile acids. GS treatment decreases hepatic cholesterol in wild-type mice fed a high-cholesterol diet but is not effective in FXR-null mice. Inhibition of FXR activation is proposed as the basis for its cholesterol-lowering activity [44, 63].

Extraction of salvia (PSME, containing Danshensu, salvianolic acid A/B, and rosmarinic acid) might also regulate the expression of FXR/LXR*α* and then induce the expression of ATP-binding cassette transporter protein family (ABCB11) and mouse Mdr2 P-glycoprotein (also known as ABCB4), which is responsible for bile cholesterol solubility and bile secretion in bile salts and bile phospholipids. The transcriptional activation experiments showed that PSME is a coagonist for FXR and LXR *α*. PSME might improve lipid spectrum of male high-fat high-cholesterol diet-induced hyperlipidemic SD rats by activating FXR/LXR *α*, and PSME decreases liver and plasma TG through an FXR-SHP-SREBP-1c pathway [41].

## 8. Conclusion

**Figure 1:**
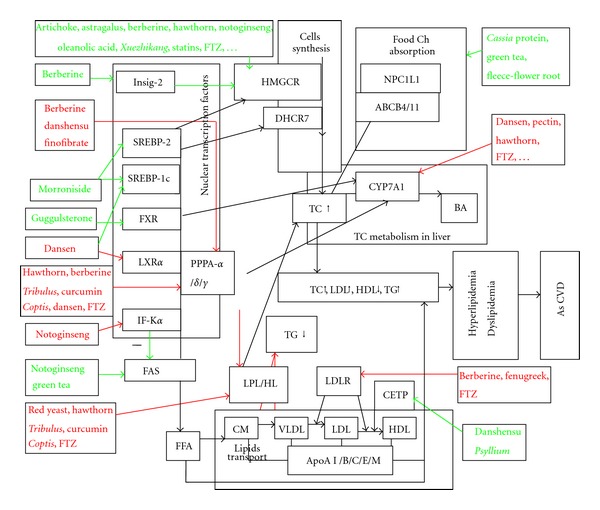
The regulation of some vital targets related to lipid metabolism by TCM. TCM might regulate all processes of lipid metabolism such as the synthesis, absorption, transport and metabolism of cholesterol and TG. Targets related to the regulation of lipid metabolism by TCM herbs are mainly including HMGCR FAS, LDL-R, CEPT, LPL, HL, CYP7A1, PPPA-*α*, SREBP, and LXRa. Some Chinese herb active components including berberine, danshensu, curcumin and hawthorn flavonoids, and ginseng saponins may regulate the different processes of lipid metabolism, and some vital targets relate to lipid metabolism as the figure arrow showed. Red arrows present positive or upregulation. Green arrows demonstrate negative or downregulation.

In summary, TCM might regulate all processes of lipid metabolism (Figure 1). Targets related to the regulation of lipid metabolism by TCM herbs are mainly HMG-CoA reductase, FAS, LDL-R, CEPT, LPL, HL, CYP7A1, PPPA-*α*, SREBP, LXRa, and other targets (Tables 1, 2, Figure 1). The Chinese herb active components include alkaloids (berberine), phenols (Danshensu, Tea Polyphenols), flavonoids (curcumin and hawthorn flavonoids), triterpenoid saponins (ginseng saponins, glycosides from Tribulus terrestris), and statins (*Monascus* prime, red yeast), which demonstrates multitarget, multicomponent features of traditional Chinese herb medicine for the regulation of lipid metabolism (Tables 1 and 2, Figure 1).

However, presently most of the studies on the mechanisms of TCM focus only on the efficacy of lipid lowering (serum lipid profile in TC, TG, LDL-C, and HDL-C) of a composite herb formula or its active ingredient and usually aim at one or two targets of lipid regulation. The interaction between the components in the composite acting on the same target is rarely involved, which failed to reflect the characteristics of the mechanism of Chinese herbal composite and the full-scale picture of Chinese medicine. It is needed to unveil the mystery of TCM and mine underground advantage of the TCM composites in dealing with the complex dyslipidemia by further study on the profound mechanism of TCM composite involving interaction among the multitargets and multicomponents.

## Figures and Tables

**Table 1:** The regulation of some vital targets related to lipid metabolism by TCM.

Herb/ingredients	Ch absorption	Ch transport (LDLR, …)	Lipid catabolism (HL, LPL)	Ch synthesis (HMG-CoA R)	BA synthesis (CYP7A1)	Others	Reference
*Ananas comosus* leaves			LPL(+)	(−)			[35]
*Astragalus, Angelica*/ferulic acid				(−)			[5, 14]
*Alpinia officinarum*/3-Methylethergalangin			Pancreatic L(−)				[34]
Artichoke/cynaroside, luteolin				(−)			[15]
Cassia protein	(−)						[6]
Coptis/berberine		(+)	LPL(+)	(−)			[32, 36–38]
Danshen/dihydrotanshinone 1, salvianolic acid, danshensu	(+)	LDLR(+), CEPT(−)		(−)	(+)	ABCA4/11(+)	[39–41]
FTZ		(+)	HL(+), LPL(+)	(−)	(+)		[32]
Fenugreek (TEFS)		(+)					[42]
Fleece-flower root/anthraquinones, Lecithin,	(−)	(+)			(+)	ACAT(−)	[5]
Garlic						MTP(−)	[12]
Ginseng			LPL(+)				[28]
Green tea/catechol, saponin	(−)					FAS(−)	[17, 18]
*Gynostemma pentaphyllum*/gypenoside				(−)			[43]
Hawthorn/hawthorn-flavone		ACAT(−)	LPL(+)	(−)	(+)	ACAT(−)	[24]
Resin of the guggul tree/guggulsterone				(−)	(+)		[44]
Hawthorn, ligustrum lucidum/oleanolic acid				(−)		ACAT(−)	[11, 51]
Notoginseng/saponin						FAS(−)	[16]
Pectin				(+)	(+)		[46]
*Pleurotus eryngii*	(−)		Pancreatic L(−)				[33]
*Plantago ovata*		CEPT(−)		(+)	(+)	LACT(−)	[48]
Psyllium		CEPT(−)		(+)	(+)	ApoB/LCAT(−)	[47]
Red yeast/xuezhikang		(+)	LPL(+)	(−)			[27]
*Tribulus terrestris*/saponins			HL(+), LPL(+)				[31]
Turmeric/curcumin			LPL(+)				[25, 26]

**Table 2:** The regulation of transcription factors related to lipid metabolism by TCM.

Herb/ingredients	PPAR-*α*/*γ*/*δ*	LXR-*α*	SREBPs	FXR	Others	Reference
Berberine	*α*(+), *γ*(+)		SRE1		Insig-i(+), CPTIA	[23, 36–38, 50]
Curcumin	*γ*(+)					[25, 26]
Danshen/dihydrotanshinone 1, salvianolic acid, and Danshensu rosmarinic acid	*α*(+)	(+)	−1c(−)	(+)	ABCA1, ABCB4/11, SHP(+)	[40, 41]
Ginseng	*α*(+)					[45]
*Gynostemma pentaphyllum*/gypenoside	*α*(+)					[43]
Resin of the guggul tree/guggulsterone				(−)	AR/GR/MR(−); PR/ER*α*(+)	[44]
*Ligustrum lucidum*/oleanolic acid	*α*(+), *γ*(+)				protein kinase B (+)	[51]
Hawthorn, hawthorn-flavone	*α*(+)					[11, 24]
Morroniside	*α*(+)		−1(−);** −**2(−)		NF-k*β*, iNOS, (−)	[52]
FTZ	*α*(+)		(−)			[49]
Fenugreek (TEFS)	*γ*(−)		−1(−)		cEBP-*α*(−)	[42]

Notes for tables: (+): positive/upregulation; (−) negative/downregulation.

## Abbreviations

ABC: ATP-binding cassette
ABCA1: ATP-binding cassette transporter A1
ABCB4: Mdr2 P-glycoprotein
ABCB11: ATP-binding cassette transporter protein family
ABCG5/8: ATP-binding cassette transporters ABCG5/ABCG8
AC: The ethanolic extract of *Ananas comosus* L. leaves
ACAT: Acyl coenzyme A cholesterol acyltransferase
ApoA/B: Apolipoprotein A/B
AR: Androgen receptors
BA: Bile acid
CETP: Cholesterol ester transfer protein
Ch: Cholesterol
c/EBP-alpha: CAAT element-binding proteins-alpha
ER*α*: Estrogen receptor alpha
CYP7A1: Cholesterol 7*α*-hydroxylase
FAS: Fatty acid synthase
FTZ: Fufang Zhenzhu Tiao Zhi
FXR: Farnesoid X receptor
GR: Glucocorticoid receptors
HDL-C: High-density lipoprotein cholesterol
HL: Hepatic lipase
HMGCR: HMG-CoA reductase
iNOS: Inducible nitrogen oxide synthase
Insig-2: Insulin-induced gene-2
LACT: lecithin cholesterol acyltransferase
LDL-C: Low-density lipoprotein cholesterol
LDLR: LDL receptor
LPL: Lipoprotein lipase
LXR*α*: Liver X receptor *α*
MR: Mineralocorticoid receptors
MTP: Microsomal triglyceride transfer protein
NF-k*β*: Nuclear factor-kappa beta
NPC1L1: Niemann-Pick C1 Like 1
PPAR*α*/*γ*/*δ*: Peroxisome proliferators activated-receptor -alpha/gamma/delta
PR: Progesterone receptors
PSME: Salvia extract
SHP: Short heterodimer partner
SRE-1: Sterol regulatory element 1
SREBP-1/2: Sterol regulatory element-binding protein-1/2
SR-B1: Scavenger receptor B1
TC: Cholesterol
TCM: Traditional Chinese medicine
TG: Triglyceride
TEFS: Thermostable extract of fenugreek (*Trigonella foenum-graecum*) seeds
VLDL: Very low-density lipoprotein.
